# Molecular Pathogenesis of Virus Infections

**DOI:** 10.3201/eid1201.051305

**Published:** 2006-01

**Authors:** Robert Buller

**Affiliations:** * St. Louis University, St. Louis, Missouri, USA

**Keywords:** Molecular Pathogenesis of Virus Infections, Paul Digard, Anthony Nash, Richard Randall

Molecular Pathogenesis of Virus Infections describes our current understanding of the pathogenesis of selected virus and prion infections. The innate response is an early barrier to virus spread. In this context, O. Haller et al. describe the antiviral activity of type I interferons and the various virus-encoded countermeasures. R.P. van Rij and R. Andino review the role of RNAi as a therapeutic antiviral agent and its use by the host and virus during viral infections. J.L. Whitton gives an overview of the adaptive CD8+ T-cell immune response in the context of virus infections. G. Screaton and J. Mongkolsapaya explain potential roles of T-cell responses in dengue hemorrhagic fever. E. Turnbull and P. Borrow provide a detailed description of the ineffectual roles of the innate and immune responses in the control of HIV and the long road ahead for development of either a prophylactic or therapeutic vaccine.

Transmissible spongiform encephalopathies have perhaps the most unconventional natural history of any infectious agent. J.C. Manson and R.M. Barron describe the diagnosis of transmissible spongiform encephalopathies, the appearance of new strains, and the nature of host susceptibility to disease. C.M. Dixon et al. depict the special problems presented to the host by certain RNA viruses that are maintained and persist in human populations through avoidance or inhibition of apoptosis, innate immune response, and adaptive immune response.

Other viruses infect humans only as incidental hosts and cause epizootics of varying degrees. A.L. Hartman et al. review our current understanding of the pathogenesis of Ebola and Marburg filoviruses, paying particular attention to the factors that contribute to lethal disease. C. Dye and S. Siddell discuss the pathogenesis of feline coronavirus, an animal disease model that has provided insights into the study of the newly recognized disease, severe acute respiratory syndrome. R.G. Webster et al. enumerate the key influenza genes responsible for human pathogenicity, their roles in past pandemics, and the potential of avian influenza virus strains to evolve into highly pathogenic and transmissible viruses for human populations.

Many viruses modify host metabolism and innate/immune responses to their own ends. L. Gray et al. describe the impact of human papillomaviruses on cell cycle and apoptosis. S.M. Lemon and K. Li review the data documenting hepatitis C virus disruption of innate intracellular antiviral defenses, including interferons and toll-like receptors. M.B. Ruiz-Arguello et al. enumerate the multiple, distinct receptor homologs and binding proteins encoded by poxviruses that target tumor necrosis factor. L.K. Dixon summarizes the multiple host pathways that are targeted at multiple levels by African swine fever virus. J.P. Stewart et al. describe the pathogenesis of murid herpesvirus 4 that supports its use as model for gammaherpesviruses. M.L. Freeman et al. provide an overview of the potential role of the immune system in the latency of the alphaherpesvirus, herpes simplex virus 1. This book is suitable for the serious student and professional and is well referenced for further reading.

